# A Comprehensive Review of Evaluating Donor Site Morbidity and Scar Outcomes in Skin Transfer Techniques

**DOI:** 10.7759/cureus.53433

**Published:** 2024-02-01

**Authors:** Yogesh B Manek, Suhas Jajoo, Chandrashekhar Mahakalkar

**Affiliations:** 1 General Surgery, Jawaharlal Nehru Medical College, Datta Meghe Institute of Higher Education & Research, Wardha, IND

**Keywords:** tissue engineering, graft characteristics, scar outcomes, donor site morbidity, skin transfer techniques, reconstructive surgery

## Abstract

This comprehensive review delves into the intricacies of donor site morbidity and scar outcomes in skin transfer techniques central to the field of reconstructive surgery. The review synthesizes existing literature to illuminate the multifaceted factors influencing outcomes by surveying a broad spectrum of grafting methods, from traditional autografts to cutting-edge tissue engineering approaches. Key findings underscore the complex interplay of graft characteristics, surgical techniques, and patient-specific variables. The implications for clinical practice advocate for a nuanced, patient-centered approach, incorporating emerging minimally invasive procedures and adjuvant therapies. The review concludes with recommendations for future research, emphasizing the importance of longitudinal studies, comparative analyses, patient-reported outcomes, advanced imaging techniques, and exploration of tissue engineering innovations. This synthesis advances our understanding of donor site morbidity and scar outcomes. It provides a roadmap for refining clinical protocols, ultimately enhancing the delicate balance between therapeutic efficacy and patient well-being in reconstructive surgery.

## Introduction and background

Reconstructive surgery has witnessed remarkable advancements, particularly in skin transfer techniques. The intricate art and science of grafting have become indispensable tools in the hands of surgeons, enabling the restoration of form and function for patients affected by trauma, burns, or congenital anomalies. In the broader context of reconstructive procedures, the significance of skin transfer techniques cannot be overstated [1]. Skin transfer techniques have evolved into a cornerstone of reconstructive surgery. These techniques serve as pivotal instruments in restoring damaged or lost skin, contributing to physical rehabilitation and the psychological well-being of patients. From autografts to advanced tissue engineering approaches, the array of available methods underscores the versatility of skin transfer techniques in addressing diverse clinical scenarios [2].

Despite the evident success of skin transfer techniques, a burgeoning concern within the field centers around the repercussions associated with donor site morbidity and scar outcomes. As the frequency and complexity of reconstructive procedures rise, so does the imperative to scrutinize and ameliorate the consequences at the donor site. Regarding physical discomfort and aesthetic considerations, the long-term impact on patients necessitates a comprehensive evaluation of these outcomes [3].

This review seeks to comprehensively explore and analyze the multifaceted aspects of donor site morbidity and scar outcomes in the context of skin transfer techniques. By synthesizing existing knowledge and scrutinizing relevant literature, the aim is to provide a nuanced understanding of the challenges posed by these outcomes and to identify areas requiring further investigation. Through this, the review intends to contribute valuable insights that can inform clinical practice and guide future research endeavors.

## Review

Factors influencing donor site morbidity and scar outcomes

Graft Thickness

The thickness of skin grafts varies depending on the graft type. Split-thickness skin grafts (STSGs) are categorized into thin (0.15 to 0.3mm), intermediate (0.3 to 0.45mm), and thick (0.45 to 0.6mm) based on their thickness [4]. For an intermediate-thickness STSG, the standard thickness is typically set at 0.015 - 0.018 inches, accommodating the width of a #15 blade [5]. Full-thickness skin grafts (FTSGs) are recommended for small avascular areas less than 1 cm or more substantial areas with a robust blood supply. In dermatology, FTSGs are the most frequently employed graft type, offering an excellent tissue match for the host site and promoting minimal scarring during the healing process [4,5]. The decision between full- and split-thickness grafting depends on the wound condition, location, thickness, size, and aesthetic considerations [4].

Graft Size

The size of a graft is contingent upon both the type of graft and the location of the graft site. For intermediate-thickness split-thickness skin grafts (STSGs), a standard thickness is typically set between 0.015 and 0.018 inches [5]. In the case of STSGs, a technique known as "pie-crusting" can be employed to manually create small perforations using a blade, which is particularly useful for larger grafts [6]. Full-thickness skin grafts (FTSGs) find indications in small avascular areas measuring less than 1 cm or more substantial areas possessing a robust blood supply [7]. In the context of anterior cruciate ligament (ACL) reconstruction, the diameter of the autograft emerges as a pivotal factor influencing biomechanical and clinical outcomes [8]. Clinical studies have extensively documented that hamstring grafts with a width of less than 8 mm are notably more susceptible to failure [8]. It becomes evident that whether in skin grafting or ACL reconstruction, the graft size is critical in determining the success of the procedure and the subsequent outcomes for the patient.

Graft Location

The selection of a donor site for a skin graft is versatile. It can encompass various body areas, with a preference for locations typically concealed by clothing, such as the buttock or inner thigh. The graft is meticulously spread over the designated area for transplantation, securing its position through gentle pressure facilitated by a well-padded dressing, staples, or a few small stitches. Following the grafting procedure, the donor site is carefully covered with a sterile dressing for 3 to 5 days to promote optimal healing. In the case of STSGs, harvesting is commonly performed from broad, flat regions, including the anterolateral thighs, back, trunk, lateral arm/forearm, and lateral lower leg. These areas are the most accessible donor sites for graft extraction when utilizing a mechanical dermatome. On the other hand, FTSGs find indications in cases involving small avascular areas measuring less than 1 cm or more extensive areas with a robust blood supply. When considering facial FTSGs, the supraclavicular, preauricular, postauricular, and inner arm emerge as common and preferred donor sites [4,9,10]. This strategic selection of donor sites aligns with functional and aesthetic considerations, contributing to the success of the grafting procedure.

Patient-Specific Factors

Age: Patient age is a pivotal patient-specific factor that can influence the outcomes of skin grafting procedures. In the context of long-term scarring outcomes and the safety of patients treated with NovoSorbR Biodegradable Temporizing Matrix (BTM), a study revealed that age did not emerge as a significant factor affecting scarring outcomes [11]. Conversely, in a study focusing on lower limb skin graft failure, it was observed that the median age of participants, particularly at 79 years old, did not serve as an identified risk factor for graft failure [12]. In the realm of anterior cruciate ligament (ACL) reconstruction, where the diameter of the autograft holds paramount importance for biomechanical and clinical outcomes, a study established a noteworthy correlation between the size of the 4-strand autograft diameter and the likelihood of requiring ACL revision surgery [7]. Beyond age, other patient-specific factors, such as tobacco use, anti-coagulant use, bleeding disorders, chronic steroid use, or malnutrition, can significantly influence the outcomes of skin grafting procedures [4]. A comprehensive understanding of these patient-specific factors is integral for optimizing wound care and making well-informed decisions regarding skin grafting techniques, ultimately contributing to improved outcomes for the patient.

Health status: A patient's health status plays a crucial role in determining the success of skin grafting procedures. A study focusing on patients with venous leg ulcers investigated the impact of split-thickness skin grafting on quality of life and self-esteem. The findings revealed that patients undergoing this grafting method experienced better health-related quality of life and enhanced self-esteem than those undergoing conservative treatment [13]. Conversely, another study examining health-related quality of life and patient burden in individuals with split-thickness skin graft donor site wounds found decreased health-related quality of life and increased patient burden following the skin grafting procedure [14]. In the context of lower limb skin graft failure, peripheral vascular disease (PVD), elevated body mass index (BMI), and the use of immunosuppressant medications emerged as significant risk factors for graft failure [12]. Beyond these factors, patient-specific variables such as tobacco use, anti-coagulant use, bleeding disorders, chronic steroid use, or malnutrition can also exert notable influences on skin grafting outcomes [4]. A comprehensive understanding of these patient-specific factors is imperative for optimizing wound care and making well-informed decisions regarding skin grafting techniques, ultimately leading to improved outcomes for the patient.

Skin type: Skin type significantly influences skincare outcomes and dermatological procedures, including skin grafting. The American Academy of Dermatology identifies five primary types of skin: oily, dry, normal, combination, and sensitive [15]. Each skin type exhibits distinct characteristics and necessitates specific care regimens and products. Understanding a patient's skin type becomes crucial in determining the most suitable approach for skin grafting and post-operative care. For instance, individuals with dry skin may require more intensive moisturization to address their skin's tendency toward dryness. On the other hand, those with oily skin may necessitate special attention to prevent excessive sebum production at the graft site. Considering a patient's skin type plays an integral role in tailoring pre- and post-operative care and optimizing the outcomes of skin grafting procedures [15]. By customizing skincare approaches based on individual skin characteristics, healthcare practitioners can enhance patient comfort and contribute to the overall success of the grafting process. Table 1 describes the factors influencing donor site morbidity and scar outcomes.

**Table 1:** Factors influencing donor site morbidity and scar outcomes

Factor Category	Specific Factors
Patient Characteristics	Age, general health, skin type, comorbidities
Graft Type and Thickness	Split-thickness vs. full-thickness grafts, graft thickness
Surgical Technique	Minimally invasive approaches, use of adjuvants
Timing of Outcome Assessment	Short-term vs. long-term assessment
Quality of Life Measures	Patient satisfaction, impact on well-being
Patient Health Status	Overall health, presence of peripheral vascular disease
Skin Type	Oily, dry, normal, combination, sensitive
Pain Management	Use of pain relief measures during and after surgery
Wound Care and Follow-up	Postoperative care, adherence to follow-up instructions
Patient Expectations and Counseling	Managing patient expectations, education, and counseling
Use of Advanced Techniques	Laser-assisted procedures, dermoscopy, ultrasound

Assessment methods for donor site morbidity and scar outcomes

Patient-Reported Outcomes

Pain: Pain is a prevalent concern associated with various medical conditions and treatments, extending to skin transfer techniques where a skin traction technique with adjustable tension is employed for treating extensive skin defects. While effective, this approach may occasionally induce pain during minor traction, and postoperative scars may manifest on the skin [16]. Pain intensity evaluation is a specific outcome in the literature for assessing split-thickness skin graft donor-site morbidity, acknowledging its significance in the overall patient experience [17]. The inadequate taking of FTSGs often leads to the development of hypertrophic scars, a common aesthetic concern for patients. It contributes to postoperative pain associated with FTSG donor site morbidity [18]. Introducing a therapeutic dimension, skin rolling, a massage technique designed to alleviate skin and connective tissue restrictions, emerges as a potential strategy to mitigate pain, enhance flexibility, and alleviate tightness. Notably, this technique can be applied around areas of scar tissue to facilitate its breakdown, fostering increased healing within soft tissues [19]. Incorporating such pain-alleviating strategies into skin transfer procedures reflects a holistic approach aimed at enhancing patient comfort and optimizing the overall outcomes of these techniques.

Itching: In evaluating patient experiences, patient-reported outcomes (PROs) related to itching play a crucial role in assessing the impact of itching on an individual's overall quality of life. A widely utilized tool for this purpose is the Patient-Reported Outcomes Information System (PROMIS) Itch Questionnaire, designed to measure itch severity quantitatively and its influence on patients' well-being [20,21]. This questionnaire has undergone rigorous development and validation processes, ensuring its effectiveness in gauging the impact of itching on the quality of life. It provides a comprehensive assessment by capturing the severity and frequency of the itch, offering valuable insights into the patient experience [21,22]. The utility of PRO measures includes numeric rating scales (NRS) and verbal rating scales (VRS), which have exhibited robust content validity and demonstrated strong correlations. This consistency enhances their value in evaluating the severity and frequency of itch, contributing to a more nuanced understanding of the patient's perspective [21]. By employing these PRO measures, healthcare professionals gain valuable tools to comprehensively evaluate and address the impact of itching on patients' lives, facilitating more patient-centered care and treatment approaches.

Satisfaction with appearance: PROs concerning satisfaction with the appearance of donor sites post-skin grafting are integral to comprehensively assess the impact on a patient's well-being. The widely employed Patient and Observer Scar Assessment Scale (POSAS) is a valuable tool in evaluating scar quality, encompassing a patient-reported component that sheds light on the patient's satisfaction with the donor site's appearance [23]. Nevertheless, it is acknowledged that a more universally accepted set of assessment tools for donor-site morbidity across studies is needed, posing a challenge to standardizing the evaluation of patient-reported outcomes for donor-site satisfaction [24]. Furthermore, specific outcomes considered in the literature encompass a range of factors such as time to healing, pain intensity, hypertrophic scarring, infections, and stiffness. These outcomes collectively contribute to shaping patients' satisfaction with the appearance of the donor site [3,25]. As these elements can significantly influence the overall patient experience, their thorough evaluation is imperative for providing a holistic understanding of donor-site outcomes and ensuring a patient-centric approach in post-skin grafting care.

Objective Measures

Scar thickness and pigmentation: Objective metrics, such as scar thickness and pigmentation, are frequently employed to evaluate the morbidity of donor sites and the outcomes of scars. Scar thickness, a critical parameter, can be precisely gauged using methodologies like ultrasound or calipers, providing accurate quantitative data [26]. Similarly, pigmentation, a significant facet of scar appearance, is commonly evaluated using colorimetry, delivering objective insights into the pigmentation characteristics of the scar [26]. The Vancouver Scar Scale is a widely utilized instrument for the comprehensive assessment of scar quality, encompassing factors such as pigmentation, vascularity, pliability, and height [27]. In a study assessing scar outcomes at donor sites after split-thickness skin grafting, the patient-oriented component of the POSAS was employed for scar quality evaluation, incorporating an assessment of pigmentation among other parameters [23]. Additionally, digital colorimetric analysis has proven effective in objectively appraising the clinical outcome of donor-site healing post-skin grafting procedures [26]. Furthermore, hyperpigmentation is a common complication observed at the donor site of split-thickness skin grafts, underscoring the significance of appraising pigmentation as a crucial objective measure of donor-site morbidity [3]. By integrating these objective measures, healthcare practitioners can obtain a comprehensive and precise evaluation of scar outcomes, facilitating well-informed decision-making and optimizing postoperative care in skin grafting procedures.

Functional outcomes are pivotal objective measures in evaluating both donor site morbidity and scar outcomes. The literature extensively evaluates specific functional outcomes, encompassing parameters like time to healing, pain intensity, hypertrophic scarring, infections, pruritus, and stiffness [17]. In a study focused on assessing scar outcomes following split-thickness skin grafting, the patient component of the POSAS was utilized. This comprehensive tool includes an evaluation of functional outcomes such as pain and itching, providing a nuanced understanding of the patient's experience [23]. A systematic literature review on split-thickness skin graft donor-site morbidity underscores the significance of assessing specific functional outcomes like stiffness and pruritus. This emphasizes the importance of using functional outcomes as objective measures in evaluating donor-site morbidity, recognizing their impact on the patient's overall well-being [17]. By incorporating these comprehensive functional assessments, healthcare professionals can gain a more holistic understanding of the impact of skin grafting procedures, facilitating informed decision-making and tailored postoperative care to optimize patient functional outcomes.

Complication rates: Objective measures of complication rates in donor site morbidity and scar outcomes play a crucial role in assessing the overall success of skin transfer techniques. Although specific complication rates are not explicitly outlined in the provided search results, the literature review and studies on donor site morbidity and scar outcomes consistently evaluate complications, including hypertrophic scarring, infections, pruritus, and stiffness [17,24]. These complications represent significant objective measures contributing to the comprehensive assessment of donor site morbidity and scar outcomes. Notably, the specific rates of complications may exhibit variability based on factors such as the type of skin transfer technique employed and individual patient characteristics. Considering these complications in the evaluation process provides valuable insights into the efficacy and safety of skin transfer techniques, enabling healthcare professionals to tailor interventions and optimize patient outcomes.

Imaging Techniques

Dermoscopy: Dermoscopy emerges as a sophisticated imaging technique for evaluating donor site morbidity and scar outcomes in skin transfer techniques. This method allows for a meticulous examination of the skin's surface, offering detailed insights into the healing process, scar formation, and pigmentation changes [23,26]. In a study focused on assessing scar outcomes at donor sites following split-thickness skin grafting in burn patients, dermoscopy was employed to evaluate the long-term scarring outcomes [23,26]. The Patient and Observer Scar Assessment Scale (POSAS) was a comprehensive tool for evaluating scar quality, encompassing pigmentation, vascularity, pliability, and height [11]. Dermoscopy is valuable for monitoring the healing process and identifying potential complications, including hyperpigmentation, itching, infections, and scarring [3]. Moreover, when coupled with other imaging techniques, such as digital colorimetric analysis, dermoscopy contributes to a more comprehensive and nuanced assessment of donor site morbidity and scar outcomes [26]. This integrative approach enhances the precision of evaluations and provides healthcare professionals with a holistic understanding of the impact of skin transfer techniques on the skin's integrity.

Ultrasound: Ultrasound is an invaluable imaging technique for assessing donor site morbidity and scar outcomes in skin transfer techniques. This imaging modality offers essential information about the thickness and vascularity of scar tissue, providing insights into the healing process and identifying potential complications [23]. In a study that evaluated the safety and scarring outcomes of patients treated with NovoSorbR Biodegradable Temporizing Matrix (BTM), ultrasound was employed to assess scar thickness, exemplifying its utility in objective measurements [11]. Moreover, ultrasound has been applied to evaluate the healing process and detect potential complications such as seroma formation and hematoma [17]. Despite its efficacy, it is acknowledged that a more universal set of assessment tools for donor site morbidity is needed across studies. This challenge emphasizes the importance of standardizing the evaluation of objective measures about donor site morbidity and scar outcomes [17]. By addressing this need, healthcare professionals can enhance the consistency and comparability of assessments, ultimately contributing to a more comprehensive understanding of the impact of skin transfer techniques on donor sites.

Photo documentation: Photo documentation proves to be a valuable imaging technique for assessing donor site morbidity and scar outcomes in skin transfer techniques. This method involves capturing photographs of the donor site at various stages of the healing process, facilitating the evaluation of healing progression and the identification of potential complications [23,27]. In a study that evaluated scar outcomes following split-thickness skin grafting, the POSAS was employed to assess scar quality, encompassing parameters such as pigmentation, vascularity, pliability, and height [11]. The strength of photo documentation lies in its versatility, as it can be used with other imaging techniques like dermoscopy and ultrasound to offer a more comprehensive assessment of donor site morbidity and scar outcomes [27]. Beyond its imaging capabilities, photo documentation is a monitoring tool for tracking the healing progression and identifying potential complications such as hyperpigmentation, itching, infections, and scarring [3]. By combining these imaging modalities, healthcare professionals can gain a multifaceted understanding of the impact of skin transfer techniques on donor sites, enabling more informed decision-making and tailored interventions for optimized outcomes.

Case studies and clinical trials

Review of Selected Studies

Comparative analysis of donor site morbidity: In a comprehensive examination of long-term patient-reported donor site morbidity following the utilization of free peroneal fasciocutaneous flap in head and neck reconstruction, this study identified hyperpigmentation, itching, infections, and scarring as prevalent complications associated with the procedure [24]. Despite these observations, the study highlighted a noteworthy challenge: the absence of universal assessment tools for each donor-site morbidity across studies, which hinders standardizing the evaluation of both donor-site morbidity and scar outcomes [24]. Another study involving 120 patients delved into donor site morbidity after the harvest of split-thickness skin grafts in Southeastern Nigeria. Confirming the reality of donor site morbidity, the investigation documented common complications such as hyperpigmentation, itching, infections, and scarring [3]. Notably, the study unveiled pigmentation issues, hyper- and hypopigmentation-and systematically recorded donor site complications, particularly among patients predisposed to forming unfavorable scars [3]. In a comparative study evaluating the donor site aesthetic outcomes between epidermal grafts and split-thickness skin grafts, this research demonstrated the potential for colorimetric measurements to assess healing outcomes objectively. The study critically compared the aesthetic outcomes of donor sites between epidermal grafts and split-thickness skin grafts [26]. Shifting the focus to a comparison between medial and lateral thigh-based flaps, a study explored donor-site morbidity rates, underscoring the necessity for further research to ascertain the optimal flap design tailored to individual patient needs [28]. In an objective assessment comparing donor-site morbidity following full and thoracodorsal nerve-preserving split latissimus dorsi flaps, this study corroborated findings from previous research, with a systematic review by Lee and Mun reporting postoperative DASH scores ranging from 7.7 to 50 following full latissimus dorsi flaps [29].

Scar assessment methodologies: Several subjective scar assessment scales have been developed to qualitatively measure scar appearance, offering both patient and observer perspectives. Examples include the Vancouver Scar Scale (VSS) and the POSAS [30,31]. These tools provide valuable insights into the qualitative aspects of scars, enabling a more comprehensive evaluation of their appearance. However, objective scar measurements are crucial to monitor changes in scar quality over time and compare treatment outcomes. These objective measurements encompass color, vascularity, thickness, relief, pliability, and surface area. Despite the development of various quantitative tools, their clinical relevance and feasibility vary, and consensus on the most appropriate evaluation method is lacking [32]. The VSS, POSAS, and Visual Analog Scale (VAS) stand out as widely utilized scar assessment tools, with the VSS evaluating vascularity, pigmentation, pliability, and height, and the POSAS incorporating subjective symptoms and basic scar evaluation [30]. A notable gap in scar assessment lies in the need for improved instructions to enhance the standardization of these scales. Furthermore, many existing methods predominantly focus on the cosmetic aspects of scars, prompting a call for a more comprehensive measure. Such a measure would integrate objective and subjective parameters, including functional impairment, infection, lymphedema, and patient-reported symptoms [33]. The Stony Brook Scar Evaluation Scale (SBSES) was introduced to assess the short-term cosmetic appearance of wounds within days to weeks after injury [34].

Critical Evaluation of Methodologies

A systematic literature review was undertaken to assess donor site morbidity and scar outcomes in skin transfer techniques, with a particular focus on split-thickness skin grafting (SSG) and epidermal grafting (EG) [17,35]. The investigation revealed that EG exhibited superior donor site outcomes, faster healing, and lower morbidity than SSG, all while maintaining comparable aesthetic results [35]. Subsequently, another study utilized a validated scar assessment tool alongside digital colorimetry to scrutinize the aesthetic outcomes of donor sites between EG and SSG [26]. The outcomes underscored that EG yielded superior aesthetic results compared to SSG [26]. Furthermore, a long-term follow-up study assessed donor site morbidity after utilizing Integra for defect coverage following radial forearm flap elevation [18]. The study emphasized that poor graft acceptance in free tissue skin grafting frequently leads to hypertrophic scars, a common esthetic concern for patients [18]. The selected studies present compelling evidence that EG offers superior donor site outcomes and lower morbidity compared to SSG while maintaining comparable esthetic results. These findings suggest that EG may be a more favorable option for skin transfer techniques concerning donor site morbidity and scar outcomes. Nevertheless, further research is imperative to solidify EG's superiority over SSG across diverse clinical settings and patient populations.

Unresolved Questions and Gaps in the Literature

Comparative long-term outcomes: Current research has delved into the nuances of outcomes among diverse skin transfer techniques; however, a notable gap exists in our comprehension of their long-term implications for donor site morbidity and scar outcomes. Many existing studies predominantly concentrate on short-term effects, highlighting the pressing need for more expansive and extended comparative studies [18]. Such investigations would serve as invaluable resources, offering critical insights into the enduring consequences of various techniques. This knowledge is paramount for making well-informed choices in reconstructive surgery and facilitating the selection of methods that align with the desired long-term outcomes. Addressing this gap in research will contribute significantly to enhancing our understanding of the sustained impact of skin transfer techniques on donor sites.

Diversity of patient populations: The existing literature may not fully encapsulate the potential variations in outcomes across diverse patient populations, posing a challenge to the generalizability of findings. A more inclusive research approach becomes imperative to address this limitation, incorporating a broad spectrum of demographic and clinical characteristics. This comprehensive strategy is essential for elucidating potential disparities in donor site morbidity and scar outcomes [35]. By encompassing a diverse range of patient profiles, this approach ensures a more nuanced understanding of the benefits and challenges associated with skin transfer techniques. Such insights are crucial for guiding personalized treatment strategies that can effectively cater to individual patient's unique characteristics and needs.

Standardized assessment tools: Even though some studies have incorporated validated tools such as the POSAS, there remains a need for improvement in standardized assessment tools. Establishing uniform assessment measures ensures consistency and comparability across studies evaluating donor site morbidity and scar outcomes [36]. The development and widespread adoption of standardized tools would play a pivotal role in facilitating more accurate comparisons, thereby enhancing the reliability of research findings in this field. This emphasis on standardization contributes to the advancement of methodological rigor in evaluating and understanding the impact of skin transfer techniques on donor sites.

Optimal timing for outcome assessment: The variability in the timing of outcome assessments across studies poses a challenge in accurately capturing both short-term and long-term effects of skin transfer techniques. Future research endeavors should focus on determining the most appropriate time points for evaluating donor site morbidity and scar outcomes to mitigate this challenge. A standardized approach to timing would enhance the precision of assessments and contribute to a more comprehensive understanding of the temporal aspects associated with these outcomes [18]. By establishing a common framework for evaluating outcomes at specific intervals, researchers can ensure a more cohesive and nuanced interpretation of the impact of skin transfer techniques over time. This standardization is vital for advancing the reliability and comparability of findings in the field.

Quality of life measures: While some studies have assessed patient satisfaction, obtaining a more holistic understanding of the impact of skin transfer techniques on patients' well-being requires incorporating comprehensive quality-of-life measures. Future research endeavors should prioritize evaluating the broader impact of these techniques on various aspects of patient's lives, offering insights beyond physical outcomes and fostering a more patient-centered approach in clinical practice [35]. Addressing these unresolved questions through well-designed clinical trials and case studies is pivotal for advancing our understanding and optimizing patient care in the context of donor site morbidity and scar outcomes in skin transfer techniques. By adopting a more comprehensive perspective that considers the broader implications for patients, clinicians can tailor interventions to enhance both physical and psychosocial aspects of patient well-being.

Strategies for minimizing donor site morbidity

Advanced Surgical Techniques

Minimally invasive approaches: Minimally invasive approaches encompass surgical techniques designed to limit the size of required incisions, thereby reducing wound healing time, associated pain, and the risk of infection [37]. Surgeons employ minimally invasive surgery (MIS) techniques and advanced technology to minimize trauma during procedures. This leads to smaller incisions, decreasing pain, lower complication rates, and faster recovery [38]. Minimally invasive surgery finds application in the diagnosis and treatment of various conditions, including cancers, abdominal issues such as appendicitis or gallbladder problems, hip and knee replacements, hiatal hernia repair, select heart surgeries, lung surgeries, and weight loss surgeries, among others [38]. Minimally invasive surgical techniques include laparoscopy, arthroscopy, endoscopy, and keyhole surgery [38]. These techniques offer a safer alternative to traditional open surgery, providing benefits such as reduced pain, shorter hospital stays, and fewer complications [38]. The minimally invasive approach has become widely adopted across different medical specialties, offering patients a more favorable experience with improved outcomes and a quicker return to normal activities.

Laser-assisted procedures: Laser-assisted procedures, particularly low-level laser therapy (LLLT), have garnered attention for their potential to reduce donor site morbidity in skin grafting techniques. A case series study focused on the effects of LLLT on both donor and recipient sites of free gingival grafts, revealing enhanced healing and reduced post-operative pain at the donor site [39]. While the study centered on gingival grafts, the findings imply the potential of LLLT in mitigating donor site morbidity in broader skin transfer procedures. A systematic literature review underscored the multifaceted nature of donor site morbidity, encompassing issues such as pain, discomfort, infections, pruritus, wound exudation, and aesthetic dissatisfaction [17]. Furthermore, a study showcased the utilization of fasciocutaneous free tissue transfer for harvesting split-thickness skin grafts, resulting in inconspicuous healing and comparable outcomes, offering a potential avenue for minimizing additional donor site morbidity [40]. These findings suggest that laser-assisted procedures, like LLLT, and specific surgical techniques, such as fasciocutaneous free tissue transfer, promise to reduce donor site morbidity in skin transfer techniques. Continued research and accumulating clinical evidence will likely provide additional insights into the effectiveness of these advanced approaches, further shaping their role in optimizing outcomes for patients undergoing skin transfer procedures.

Use of Adjuvants

Topical treatments: The exploration of adjuvants, notably platelet-rich plasma (PRP), to mitigate donor site morbidity in skin grafting has been studied. However, existing evidence does not strongly endorse the use of PRP as an adjuvant therapy in various contexts, including skin grafts, burns, carpal tunnel surgery, or scars. A study proposed that while PRP may potentially enhance split-thickness skin graft donor site healing, the overall morbidity from the donor site remains low, posing a challenge in justifying the use of PRP from a health economics perspective [41]. Moreover, a systematic literature review on split-thickness skin graft donor-site morbidity emphasized the diverse outcomes under evaluation, ranging from time to healing and pain intensity to hypertrophic scarring, infections, pruritus, and stiffness [17]. Additionally, another study underscored the real complications associated with donor sites of split-thickness skin grafts, including hyperpigmentation, itching, infections, and scarring, contributing to the overall donor site morbidity [3]. While the use of adjuvants, particularly PRP, has been explored, the current evidence and practical considerations based on health economics suggest the necessity for further research and evaluation of adjuvant therapies to establish their effectiveness in minimizing donor site morbidity in skin transfer techniques. Continued investigation is vital for informing clinical decision-making and optimizing patient outcomes.

Pharmacological interventions: The investigation of adjuvants, especially pharmacological interventions, aimed at minimizing donor site morbidity in skin transfer techniques has been a topic of study. However, current evidence does not robustly support using specific adjuvants, such as platelet-rich plasma (PRP), in various contexts like skin grafts, burns, carpal tunnel surgery, or scars. The potential benefits of PRP, even in expediting split-thickness donor site healing, are challenged by the overall low morbidity from the donor site, creating difficulties in justifying its use based on health economics considerations [41]. Donor site morbidity in skin transfer techniques encompasses a range of outcomes, including pain, discomfort, infections, pruritus, wound exudation, and aesthetic dissatisfaction [40]. Therefore, while exploring adjuvants, such as PRP, has been undertaken, the existing evidence and practical considerations grounded in health economics suggest the imperative for further research and evaluation of adjuvant therapies to establish their effectiveness in minimizing donor site morbidity in skin transfer techniques. Ongoing inquiry in this domain is essential for informed clinical decision-making and optimizing patient outcomes.

Patient Education and Counseling

Managing expectations: Patient education and counseling are pivotal in effectively managing expectations and minimizing donor site morbidity in skin transfer techniques. Through comprehensive information and guidance, healthcare professionals can empower patients to understand potential outcomes, thereby reducing postoperative complications. Donor site morbidity encompasses a spectrum of aspects, including pain, discomfort, infections, pruritus, and aesthetic dissatisfaction. Therefore, educating patients about these potential challenges and offering counseling on postoperative care and follow-up can significantly contribute to managing their expectations and optimizing the recovery process. Within the context of donor site morbidity, specific outcomes under evaluation include time to healing, pain intensity, hypertrophic scarring, infections, and stiffness [17]. Moreover, studies have underscored the importance of sharing donor outcome reports with prospective donors, providing them with essential information and insights into potential morbidity considerations [42]. Additionally, research has introduced methods to reduce donor-site morbidity in specific procedures, highlighting the significance of tailored approaches and the integral role of patient education [43]. In essence, patient education and counseling emerge as integral components in the concerted effort to minimize donor site morbidity in skin transfer techniques. Equipping patients with essential knowledge and support, healthcare professionals foster more informed decision-making and ultimately improve postoperative outcomes.

Postoperative care instructions: Patient education and counseling regarding postoperative care instructions are indispensable strategies for minimizing donor site morbidity in skin transfer techniques. Furnishing patients with comprehensive information and guidance on wound care, pain management, and follow-up care is instrumental in managing their expectations and reducing postoperative complications. Donor site morbidity encompasses diverse outcomes, including pain, discomfort, infections, pruritus, and aesthetic dissatisfaction [17]. Thus, educating patients about these potential challenges and counseling them on postoperative care and follow-up significantly contributes to managing expectations and optimizing the recovery process. Studies emphasize the importance of sharing donor outcome reports with prospective donors to provide essential information and insights into potential morbidity considerations [42]. Additionally, research introduces methods for reducing donor-site morbidity in specific procedures, underscoring the significance of tailored approaches and patient education [44]. A systematic literature review on split-thickness skin graft donor-site morbidity further highlights the outcomes evaluated, encompassing time to healing, pain intensity, hypertrophic scarring, infections, pruritus, and stiffness [17]. In essence, patient education and counseling on postoperative care instructions are integral components of the holistic approach to minimizing donor site morbidity in skin transfer techniques. Healthcare professionals foster more informed decision-making and ultimately enhance postoperative outcomes by providing patients with the necessary knowledge and support.

## Conclusions

In conclusion, this comprehensive review has provided a thorough examination of donor site morbidity and scar outcomes in skin transfer techniques within the realm of reconstructive surgery. The synthesis of diverse literature has uncovered key findings, revealing the intricate interplay of factors influencing these outcomes, ranging from graft characteristics and surgical techniques to patient-specific variables. The implications for clinical practice are profound, urging practitioners to adopt a holistic and personalized approach in light of evolving surgical techniques and the potential benefits of minimally invasive procedures and adjuvant therapies. Furthermore, the review offers a set of recommendations for future research endeavors, emphasizing the need for longitudinal studies, comparative analyses, incorporation of patient-reported outcomes, exploration of advanced imaging techniques, and a focus on emerging tissue engineering innovations. By addressing these research avenues, the scientific community can contribute to refining clinical protocols, enhancing patient outcomes, and advancing the field of reconstructive surgery, maintaining a delicate equilibrium between therapeutic efficacy and patient well-being.
